# Simultaneous laparoscopic total extraperitoneal inguinal hernia repair and laparoscopic appendectomy for Amyand’s hernia: a case report

**DOI:** 10.1186/s13256-019-2131-7

**Published:** 2019-06-26

**Authors:** Daisuke Muroya, Shinji Sato, Masayuki Okabe, Yukiya Kishimoto, Keiichiro Tayama

**Affiliations:** Department of Surgery, Munakata Suikokai General Hospital, 1-7-5 Himakino, Fukutsu, Fukuoka prefecture 811-3207 Japan

**Keywords:** Amyand’s hernia, TEP, Mesh

## Abstract

**Background:**

An Amyand’s hernia is defined by the presence of a vermiform appendix within an inguinal hernia sac. Most of these cases are not diagnosed preoperatively and the surgical approach is dependent on the type present and associated intraoperative findings. We present a case of a preoperatively diagnosed Amyand’s hernia in a man who underwent treatment by simultaneous laparoscopic totally extraperitoneal repair and laparoscopic appendectomy.

**Case presentation:**

We encountered the case of a 76-year-old Japanese man with a right inguinal pain. Ultrasound and computed tomography confirmed his vermiform appendix herniated into the right inguinal canal. We managed a simultaneous laparoscopic total extraperitoneal inguinal hernia repair with mesh and laparoscopic appendectomy. He was discharged without any postoperative morbidity.

**Conclusions:**

We recommend laparoscopic appendectomy and totally extraperitoneal hernia repair with mesh after laparoscopic reduction for Amyand’s hernia.

## Background

A vermiform appendix located within an inguinal hernia sac is termed Amyand’s hernia (AH); Claudius Amyand reported a case of a perforated appendix in an inguinal hernia sac in 1735 [1]. The incidence of an appendix within an inguinal hernia is seen in 0.1% of all inguinal hernias, and the diagnosis is usually made intraoperatively [2]. Therefore, most of these cases are managed during surgery. We present a case of a preoperatively diagnosed Amyand’s hernia (AH) in a man who underwent treatment by simultaneous laparoscopic totally extraperitoneal (TEP) repair and laparoscopic appendectomy.

## Case presentation

**Fig. 1:**
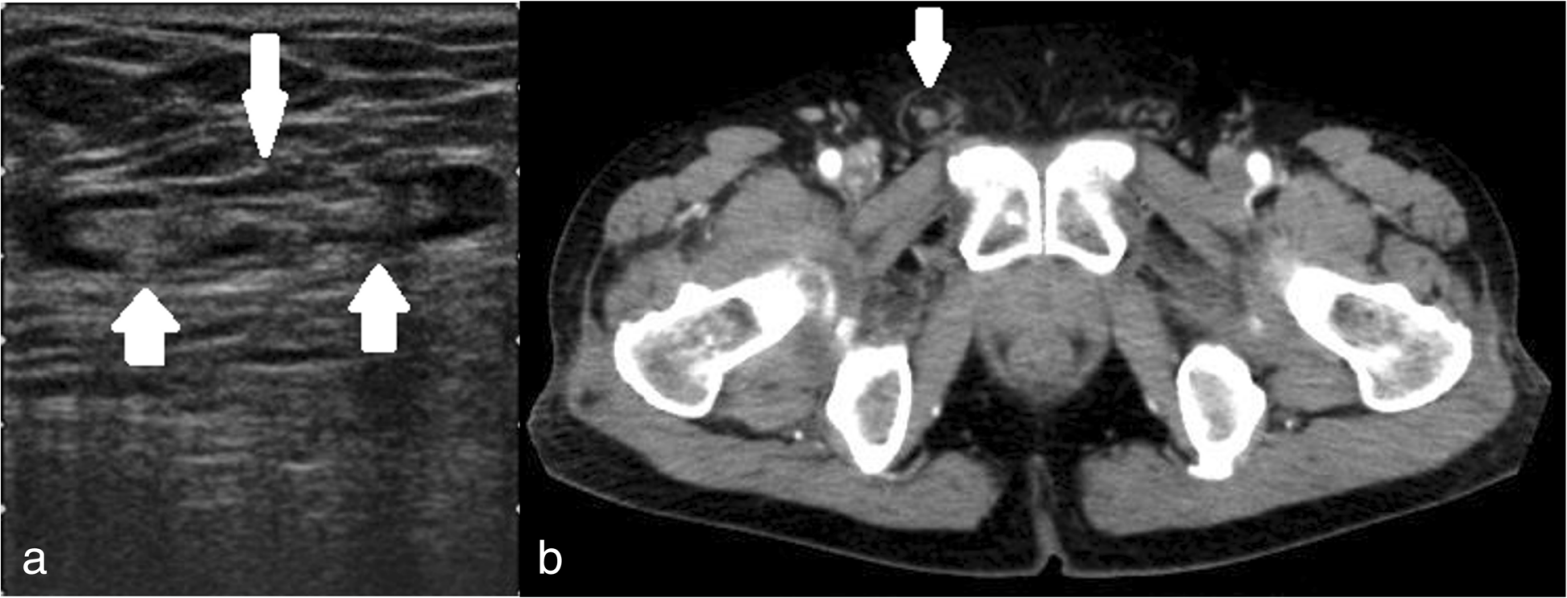
Preoperative imaging. **a** Ultrasonography showing non-inflamed appendix (arrow) inside the right inguinal canal. **b** Axial computed tomography (CT) scan showing non-inflamed appendix (arrow) in right inguinal hernia canal

A 76-year-old Japanese man was referred to our department with a several-week history of right inguinal pain and discomfort in his right femur that worsened with movement. Laboratory tests showed a normal white blood cell count and C-reactive protein level. Ultrasound and computed tomography examinations indicated a vermiform appendix in an inguinal hernia sac, with no remarkable findings of inflammation in the appendix (Fig. 1a, b). He was clinically diagnosed as having an AH without appendicitis. Reduction of the hernia was attempted under ultrasound but was unsuccessful. Thus, we planned combined TEP with mesh repair and laparoscopic appendectomy after laparoscopic reduction.

**Fig. 2:**
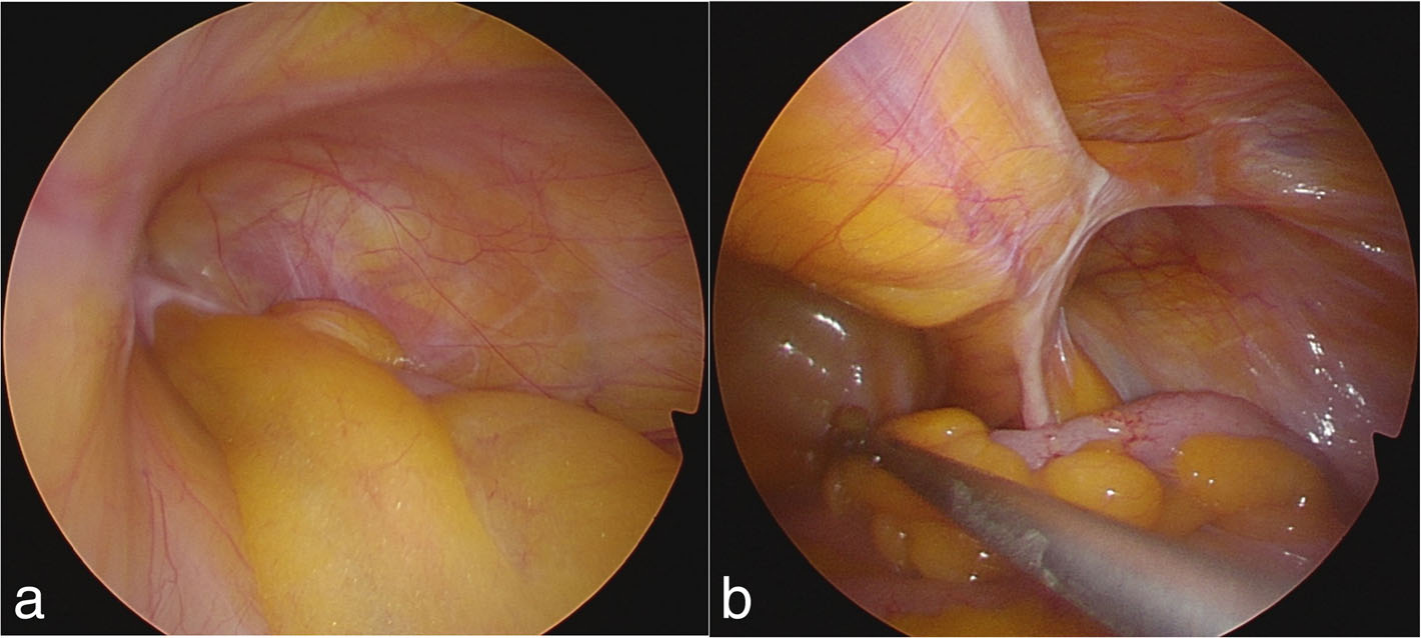
Intraoperative findings. **a** Appendix located within an external inguinal hernia canal. **b** Normal appearance of the appendix having successfully reduced from the inguinal canal, no adhesions between the vermiform appendix and surrounding hernia sac

He was placed in a supine position and underwent general anesthesia by tracheal intubation. A laparoscopic transabdominal approach was initially performed after establishment of pneumoperitoneum. A 5-mm direct umbilical trocar and a needle forceps (Endo Relief™; Hirata Precisions, Chiba, Japan) were introduced into the upper right abdominal quadrant to inspect the hernia canal for the absence of appendicitis and reduce the appendix laparoscopically (Fig. 2). This inspection revealed a 3 × 2 cm right external inguinal hernia defect with the appendix; no other intra-abdominal pathology was identified. The vermiform appendix was pulled out and placed in the abdominal cavity without tearing the appendix (Fig. 2). Next, the hernia sac was reduced into the abdomen via the laparoscopic TEP approach. Our patient was placed in the 30° Trendelenburg position. The rectus muscle was lateralized and a Covidien Balloon Dissector (Medtronic, Minneapolis, MN, USA) was inserted preperitoneally from the umbilical incision of the skin to the symphysis pubis. The balloon was insufflated to open the extraperitoneal area. Additional trocars were introduced as follows: a 12-mm trocar in the initial umbilical incision of the skin and anterior right fascia of the rectus, a 5-mm trocar at the symphysis pubis in the midline, and a 5-mm midline trocar between the symphysis pubis trocar and the umbilical trocar. To cover the myopectineal orifice, Hesselbach’s area, and the femoral canal orifice, a 7.9- × 13.4-cm mesh (3DMax™ mesh; Bard, Murray Hill, NJ, USA) was fixed to Cooper’s ligament and the rectus muscle with an absorbable fixation device (AbsorbaTack™; Medtronic). Finally, we removed the trocars and newly inserted two 5-mm trocars at the umbilical region for the intraperitoneal operation with the initial use of needle forceps. The appendectomy was completed via a laparoscopic approach, and the appendix was removed in a sterile bag via the umbilical region. The total estimated blood loss was 5 mL, and the total operation time was 111 minutes. Our patient was started on intravenously administered cefmetazole at 2.0 g intraoperatively. A histopathological examination confirmed chronic appendicitis with fibrosis and inflammatory cells. Postoperatively, he was discharged and had an uneventful recovery. He was followed up at 6 months postoperatively. He had no recurrence of the hernia, and the wound had healed without inflammatory signs.

## Discussion and conclusions

AH is a rare condition, and the diagnosis is usually made incidentally during surgery. With the widespread use of helical computed tomography in current practice, however, several authors have recently reported the ability to diagnose AH by preoperative imaging [3–5]. Surgical treatment of AH requires both appendectomy and hernia repair. The treatment algorithm for AH (Table 1) is generally accepted and recommends different management strategies depending on the severity of the condition of the appendix [6]. AH of type 3–4 is considered to be complicated by appendicitis and requires surgical treatment with avoidance of mesh. However, the efficacy of combining appendectomy and inguinal hernia repair with or without mesh for other types of AH (type 1–2) remains unclear. Some reports have described appendectomy for inflamed appendices (type 2) combined with mesh inguinal hernia repair [7–12]. Therefore, some authors consider that tension-free inguinal hernia repair with mesh and appendectomy is acceptable for both non-inflamed and inflamed appendices [3, 8, 10, 12]. In addition, Kose *et al*. [13] proposed using the presence of fibrous connections between the vermiform appendix and the surrounding hernia sac as an indicator for performing appendectomy with mesh inguinal hernia repair. Regarding the treatment of AH, several authors have suggested that laparoscopy can be a safe method for reduction of the appendix without contamination of the inguinal canal and allows the physician to rule out other pathologies [12, 14]. Mullinax *et al.* [14] published a report of a type 2 AH treated by laparoscopic hernia repair and appendectomy. Only a single report of endoscopic total extraperitoneal management of an intraoperatively diagnosed AH (type 2) has been published [15].

**Table 1:** Classification systems for Amyand’s hernia [6]

Types	Salient features	Surgical management
Type 1	Normal appendix	Reduction or appendectomy(depending on age), mesh hemioplasty
Type 2	Acute appendicitis localized in the sac	Appendectomy through hernia, endogenous repair
Type 3	Acute appendicitis, peritonitis	Appendectomy through laparotomy, endogenous repair
Type 4	Acute appendicitis, other abdominal pathology	Appendectomy, diagnostic workup and other procedures as appropriate

We performed preperitoneal mesh placement and total laparoscopic appendectomy after reducing the appendix by an intraperitoneal approach to treat a preoperatively diagnosed AH. This process was introduced to allow inspection of the hernia canal and confirm the absence of a perforated appendix or peritonitis, as well as observe the degree of fibrous connections between the vermiform appendix and the surrounding hernia sac, which helped to avoid tearing the appendix. The main reasons for selecting TEP repair are that the procedure is not influenced by intra-abdominal conditions and avoids entering the peritoneal cavity, thus protecting the mesh from bacterial contamination.

In conclusion, a laparoscopic mesh inguinal hernia repair combined with laparoscopic appendectomy can be performed for the surgical treatment of AH type 1 and select cases of AH type 2. It may be regarded as a safe technique with minimal morbidity to the patient. In particular, TEP repair of an inguinal hernia with mesh after laparoscopic hernia reduction may help to avoid mesh contamination in patients with an AH.

## Data Availability

Not applicable.

## Abbreviations

AH: Amyand’s hernia
TEP: Totally extraperitoneal
